# A giant fibroadenoma in a mature woman: diagnosis and treatment in a limited resource environment (a case report)

**DOI:** 10.11604/pamj.2021.38.19.26200

**Published:** 2021-01-08

**Authors:** Médard Kakule Kabuyaya, Fabrice Lele Mutombo, Francine Mbonga Moseka, Kasereka Kihemba, Neil Wetzig, Justin Paluku Lussy

**Affiliations:** 1 Department of Surgery, HEAL Africa Tertiary Hospital, Goma, Democratic Republic of Congo,; 2 Department of Surgery, University of Goma (UNIGOM), Goma, Democratic Republic of Congo,; 3 Familly Medicine, HEAL Africa Tertiary Hospital, Goma, Democratic Republic of Congo,; 4 Department of Pathology, HEAL Africa Tertiary Hospital, Goma, Democratic Republic of Congo,; 5 Department of Gynecology and Obstetrics, HEAL Africa Tertiary Hospital, Goma, Democratic Republic of Congo,; 6 Department of Obstetrics and Gynecology, University of Goma (UNIGOM), Goma, Democratic Republic of Congo

**Keywords:** Giant, fibroadenoma, mature woman, management, case report

## Abstract

We report an extremely rare case of a 40-year-old woman with a giant fibroadenoma of 30cm in diameter that was accompanied by ulceration and bleeding. We document the onset, the clinical presentation, as well as the challenge encountered in the diagnosis and managing in a limited resource environment.

## Introduction

Fibroadenoma is the most common type of benign lesions diagnosed in young women. A fibroadenoma is referred to a 'giant' when it is greater than 5cm, more than 500g or replaces more than 80% of the breast [1]. Fibroadenomas are typically found in women in the 2^nd^ and 3^rd^ decade of life. The prevalence in this age group has been estimated as 2.2% [2]. Giant fibroadenomas are rare representing less than 4% of all fibroadenomas. They present as a rapidly growing unilateral mass which is well circumscribed. Histologically the tumor is composed of ducts and fibrous connective tissue and can be treated with simple enucleation [3]. The differential diagnosis of giant fibroadenoma includes Phyllodes tumor, virginal breast hypertrophy (juvenile macromastia), inflammatory processes and benign proliferative lesions [4,5].

Due to the lack of specific clinical guidelines for its correct diagnosis and treatment, the management of breast masses may pose difficulties. This case report is about an extremely rare case of a 40-year-old woman with a giant fibroadenoma who was successfully managed at HEAL Africa Tertiary Hospital in the Democratic Republic of Congo (DRC). This report aims at showing that diagnosis and successful surgical treatment of such a rare case are possible in a low resource environment. It therefore contributes to the existing literature on management of giant fibroadenoma. This work has been reported according to the SCARE criteria [6].

## Patient and observation

A 40-year-old woman, nulliparous, was referred from a health centre located at nearly 350kms away from HEAL Africa Tertiary Hospital, in North Kivu province, in the Democratic Republic of Congo. The reason for referral was management of left breast cancer. She reported on May 20^th^, 2020 with complaint of a moderate painful massive tumour that had developed quite rapidly over the previous 12 months. She attempted traditional treatments including scarifications/cuts 'by the traditional healer' with no improvement in terms of reduction in size of the breast tumour. At the referring health centre, no procedure was reported. She also reported to have had episodes of intermittent right breast pain which was occasionally severe enough to interfere with her daily activities and also disturbed her sleep. She had no past medical history of any chronic illness such as hypertension, diabetes and asthma. There was no history of any medication taken on a long term basis. She had no known family history of breast or ovarian cancers. Menarche was at 12 years and she used to bleed for 3 days in a 28 days regular cycle. There was no history of painful menstruation or passage of clots during menstruation. The patient had never had a pregnancy. She did not report any history of hormone therapy. In 2012 she underwent an exploratory laparotomy for torted ovarian cyst and uterine fibroids. She did not report any history of chest wall radiation or thoracic trauma. There was no history of using oral contraceptives. She was single living as a local business lady. She neither smoke cigarette nor drink alcohol.

**Figure 1:**
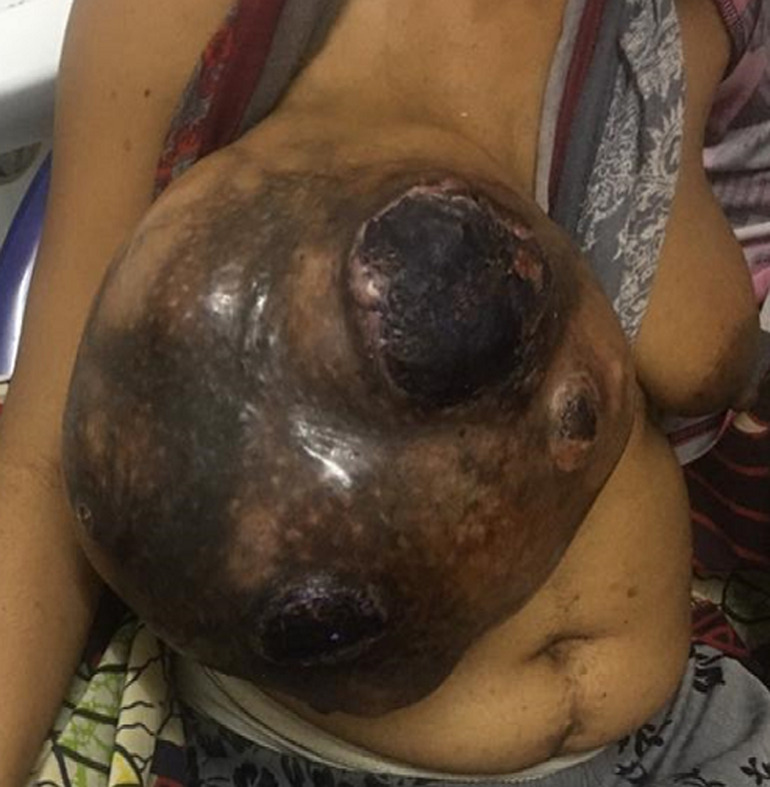
right breast tumor case before surgery

**Figure 2:**
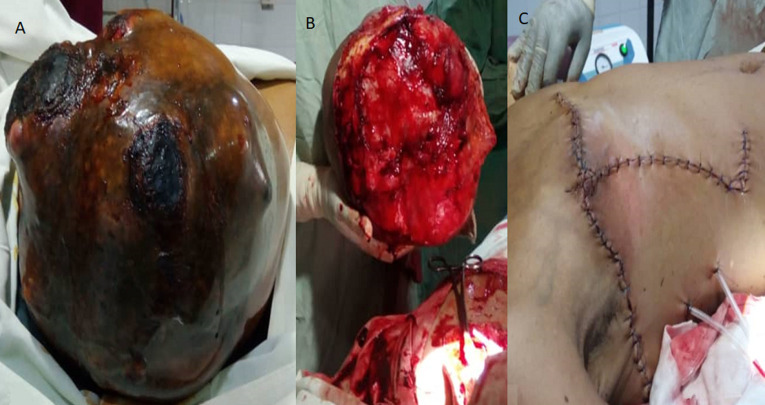
A) breast tumour preoperatively; B) right mastectomy; C) immediate postoperative aspect

General examination revealed an exhausted, pale, weak and tachycardic woman (pulse of 132 per minute). She was in painful distress. The right breast was enormously enlarged. The clinical measurement of the enlarged breast was approximately 30cm by 20cm (Figure 1, Figure 2A). There was associated slough and necrotic tissue over the ulcer with occasional contact bleeding. No axillary or supraclavicular lymphadenopathy or swelling in any other part of her body. The examination of the left breast was normal. The Initial clinical diagnosis was phyllodes breast tumor with possible differential diagnosis of fibroadenoma and adenocarcinoma of the breast. Investigations revealed normal biochemistry but severe anemia (Hb: 7.4 mg/dl; Hct: 22.1%) and a normal chest x ray. No CT-scan was available. The woman was adequately resuscitated with intravenous fluid and packed red blood cell transfusion. Intravenous ceftriaxone 1g twice daily and metronidazole 500mg 3 times a day were administered prior to surgery.

**Figure 3:**
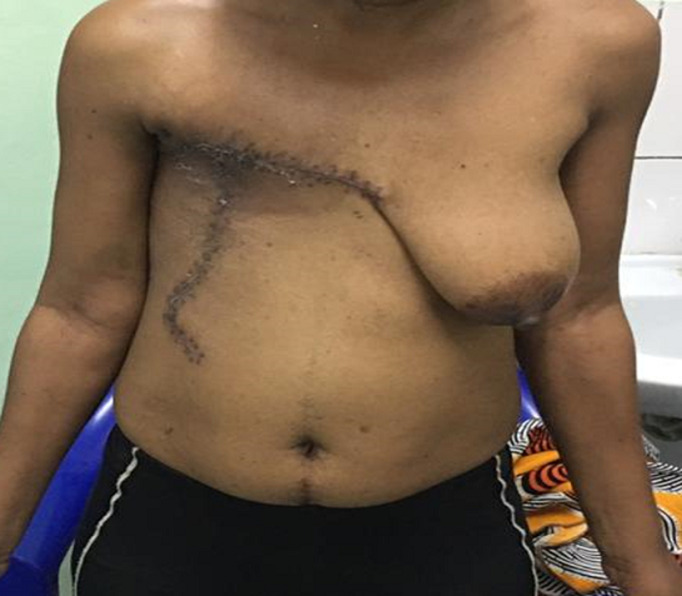
outcome after right mastectomy

After counselling and written consent of the patient a decision was made to proceed to a total mastectomy and node sampling of some enlarged lymph nodes in the axilla. Under general anaesthesia, with aseptic technique a thoraco-axillary incision was made. The breast was excised completely with ligation of the grossly dilated blood vessels. Meticulous haemostasis was achieved (Figure 2B). Two tubular drains were left in situ and the closure with interrupted suture was performed with 3-0 vicryl (Figure 2C). Macroscopically, the large masse measured at 28 X 25 cm with a weight of 4550gr. Post-surgical histopathological examination of the amputated breast revealed a giant fibroadenoma with some lymph nodes in the tail of the breast showing a non-specific reactive lymphadenitis. The skin and margins were clear of any pathology. The patient was closely monitored in ICU for 24 hours. Pain was adequately controlled and parenteral antibiotics were given. She benefited from 2 units of full blood. She had an uneventful post-operative period. Drains were removed on day 2 post-surgery and she was discharged home on day 3. The patient is on regular follow-up and doing well at two months follow-up (Figure 3). The patient is on regular follow-up and doing well at two months follow-up.

## Discussion

Fibroadenomas are generally found in women aged less than 30 years. They are classically painless, unilateral, solid and non-cancerous breast tumors [7]. Clinically, cases of giant fibroadenomas in adult woman are very rare. In the PubMed database we only found 2 reported cases. Diagnosis of a giant fibroadenoma can be difficult. When presented with a huge breast tumour, the clinicians tend to first think of a cancerous process. A thorough medical history and physical examination are of paramount value to make the diagnosis. Age of the patient and family history of breast cancer must be well documented. Literature shows that fibroadenoma are common between 14 and 35 years [7], as well as in pregnant and lactating women [8-10]. The case we are reported on occurred in a 40-year-old woman, making it an extremely rare situation. Two similar cases have been reported in a 43-year-old woman in Bulgaria in 2002 [11] and in a 39-year-old lady in Japan in 2019 [12].

Classically fibroadenomas are mobile, non-tender [13,14] with regular borders and do not show rapid growth in size [7]. The non-classical presentation of the lump in our patient was challenging as it had irregular borders with ulcerations and scars. The patient also reported moderate pain in the left breast that had lasted a month prior to admission. In Sosin M *et al*. series, about 10% of patients with fibroadenomas experienced pain [1]. The pain in our case could be the result of ulceration and necrosis occurring in the mass and resultant stretching and thinning of the surrounding skin. In fact, the more the mass increases, the tighter the skin and the more the cutaneous blood supply is compromised. Also scars due to tradition healer´s cuts could have contributed to the pain. Aetiology of fibroadenoma is debatable. It has been hypothesised that causes could be hormonal and genetic. Increased sensitivity to oestrogens may explain why the mass develops mainly during puberty around the time of menarche. Fibroadenoma may enlarge and become giant during pregnancy or lactation, in associated with an increase in oestrogens, progesterone and prolactin depending on their physiological states [7-10]. It is also hypothesised that mediator complex subunit 12 (MED12) gene plays a role in the pathophysiology of fibroadenomas [15].

Investigations are important to make the diagnosis of a fibroadenoma. Diagnostic mammogram for woman aged more than 35 and ultrasound in those who are younger are very helpful to document features of fibroadenomas [7]. In our setting, only ultrasound was available but the mass was too giant so that there was no point to request it. When diagnosis is confirmed and the mass is small, no treatment is required unless the patient wishes to have the mass excised. Fibroadenoma may diminish in size, especially after menopause. In cases where the lump is large or increasing in size, surgical removal is advised. The size of the tumor appears to be main factor that determines surgical options. Lumpectomy or excisional biopsy should be performed in this situation (cryo-ablation has been described but is seldom if ever recommended in the developing world) [7]. The present case posed a diagnostic challenge initially since the breast swelling was reported to be of short-duration (one year), no other examinations such as hormonal assays, tumor markers and certain imaging exams etc. could be carried out because the hospital is in an environment with limited resources. In the cases of suspected giant fibroadenoma, complete excision of the mass is indicated for macroscopic and histological examination. Pathological studies help to rule out differentials mainly phyllodes tumor, malignant phyllodes tumor and cancer, as clinical and radiological studies are not sufficient to do so [9]. If possible, surgery when the mass is not very large, should spare healthy breast tissue and the nipple-areolar complex [16]. Mastectomy as a treatment modality for giant fibroadenomas has been debated but is commonly reserved for unusual or recurrent cases [17].

In our case, the tumor had involved almost all of the breast tissue, coupled with extensive skin necrosis and the destruction of the entire nipple-areola complex, so we elected to perform a total mastectomy. Due to the rarity of the disease, there is no expert consensus to guide clinical practice. We therefore reviewed associated literatures to provide insights and experiences for better clinical management. The entire mass was sent to the pathologist for analysis. Histology report of a fibroadenoma describes a proliferation of both stromal and epithelial components, arranged into either a pericanalicular pattern (stromal proliferation around epithelial structures) [18]. In our case, the report is in line with fibroepithelial elements with a well-delineated capsule.

## Conclusion

Giant fibroadenomas are a rare clinical entity. Their diagnosis and successful surgical removal are possible in low resource settings. Clinicians should consider it as a differential in the presence of a massive breast lump that does not show obvious clinical signs of malignancy. But huge masses growing rapidly inside the breast can cause pressure atrophy of the surrounding normal breast tissue with subsequent ulceration of the overlying skin due to impaired blood supply thus mimicking a malignant lesion of the breast.
